# Moderate-to-Vigorous Intensity Physical Activity and Risk of Incident Atrial Fibrillation in Atrial Cardiomyopathy

**DOI:** 10.1016/j.jacadv.2026.102999

**Published:** 2026-08-28

**Authors:** Min Kim, Pil-Sung Yang, Ho-Gi Chung, Juntae Kim, Daehoon Kim, Han-Joon Bae, Moon-Hyun Kim, Boyoung Joung

**Affiliations:** a Division of Cardiology, Department of Internal Medicine, Kangbuk Samsung Hospital, Sungkyunkwan University School of Medicine, Seoul, Republic of Korea; b Division of Cardiology, Department of Internal Medicine, Yongin Severance Hospital, Yonsei University College of Medicine, Yongin, Republic of Korea; c Division of Cardiology, Department of Internal Medicine, Severance Cardiovascular Hospital, Yonsei University College of Medicine, Seoul, Republic of Korea; d Division of Cardiology, Department of Internal Medicine, Dankook University College of Medicine, Cheonan, Republic of Korea; e Division of Cardiology, Department of Internal Medicine, Daegu Catholic University School of Medicine, Daegu, Republic of Korea; f Division of Cardiology, Department of Internal Medicine, Ewha Womans University College of Medicine, Seoul, Republic of Korea

**Keywords:** accelerometer, atrial cardiomyopathy, atrial fibrillation, physical activity, UK Biobank

## Abstract

**Background:**

Current guidelines recommend physical activity for atrial fibrillation (AF) prevention, yet its role in individuals with atrial cardiomyopathy (AtCM) remains unclear.

**Objectives:**

We investigated the association between accelerometer-measured moderate-to-vigorous intensity physical activity (MVPA) and incident AF according to AtCM status.

**Methods:**

Among 10,003 UK Biobank participants, AtCM was identified via multimodal approach integrating cardiovascular magnetic resonance imaging and 12-lead electrocardiography. Multivariable adjusted Cox proportional hazards models assessed the association between 7-day accelerometer-measured MVPA and incident AF. An interaction term evaluated substrate-specific associations.

**Results:**

AtCM was identified in 4.5% of participants. Over a median follow-up of 3.2 years, AtCM was associated with an increased risk of AF (HR: 3.01; 95% CI: 1.69 to 5.37). AtCM status significantly modified the MVPA-AF association (*P* for interaction = 0.029). Without AtCM, MVPA showed marginal risk reduction (HR per 10-minute increase: 0.94; 95% CI: 0.87-1.01). Conversely, with AtCM, MVPA was strongly associated with a lower AF risk (HR per 10-minute increase: 0.76; 95% CI: 0.62-0.93). The absolute risk reduction was 2.82 AF events per 1,000 person-years for every 10-minute increase in MVPA in the AtCM group, significantly greater than without AtCM (difference: −2.66; 95% CI: -4.32 to −0.73; *P* = 0.011). Findings remained robust across comprehensive sensitivity analyses.

**Conclusions:**

MVPA demonstrated a robust inverse association with incident AF risk, particularly in individuals with AtCM. Identifying subclinical atrial substrate may guide targeted physical activity recommendations, suggesting MVPA as a potent lifestyle intervention to attenuate the transition from subclinical remodeling to overt clinical arrhythmia.

Atrial cardiomyopathy (AtCM) is increasingly recognized as a key upstream contributor to atrial fibrillation (AF), encompassing a complex spectrum of structural, functional, and electrical abnormalities that create a vulnerable substrate for arrhythmia.^1^^,^^2^ Accordingly, identifying this substrate early and validating therapeutic strategies that may attenuate the transition from subclinical remodeling to overt clinical AF is crucial.

Currently, multimodality diagnostic approaches, including 12-lead electrocardiography (ECG), echocardiography, cardiac computed tomography, and cardiovascular magnetic resonance (CMR) imaging, are used for the early AtCM identification,^3^, ^4^, ^5^, ^6^ and several associated risk factors have been reported.^7^^,^^8^ However, from a therapeutic or preventive standpoint, effective strategies to mitigate AtCM-associated arrhythmogenic risk remain poorly defined. Although physical activity is associated with reduced risk of AF in the general population^9^^,^^10^ and AF recurrence in diagnosed patients,^11^ its specific impact on an established diseased atrial substrate remains unclear. The physiological adaptations to physical activity are generally benign in healthy hearts, whereas the impact may differ in atria already compromised by fibrosis or mechanical dysfunction.

To address this uncertainty, we assessed the association between physical activity and incident AF, specifically focusing on the potential role of moderate-to-vigorous intensity physical activity (MVPA) in individuals with AtCM. We sought to determine whether the underlying atrial substrate status differentially modifies the association between MVPA and AF risk. Using the UK Biobank cohort, we identified AtCM through a multimodal integration of structural and functional phenotypes derived from CMR imaging and electrical phenotypes from 12-lead ECG. By leveraging 7-day wrist-worn accelerometer to capture objective, high-fidelity MVPA, this study aimed to provide observational insights to inform targeted, substrate-specific physical activity recommendations for AF prevention.

## Methods

### Availability of data and materials

The data that support the findings of this study are available from the UK Biobank project site, subject to the registration and application processes.

### Study population

The UK Biobank is a large-scale prospective cohort study in which over 500,000 participants aged 37 to 73 years were recruited from 22 assessment centers across the United Kingdom between 2006 and 2010. The detailed study protocol has been published previously.^12^ From the total cohort, we identified participants (n = 14,376) who had valid 7-day wrist-worn accelerometer data collected between 2013 and 2015^13^ and who underwent both CMR imaging and 12-lead ECG as part of the imaging substudy introduced in 2014.^14^ The median interval between accelerometer measurement and imaging assessment was 2.97 years.

Participants were excluded sequentially based on the following criteria: 1) insufficient accelerometer wear time (<72 h) or poor calibration (n = 896); 2) missing covariates (n = 186); 3) prevalent AF at baseline (n = 27); 4) left ventricular (LV) systolic dysfunction (LV ejection fraction <50%, n = 556); or 5) history of cardiovascular disease or major risk factors, defined as a history of heart failure, coronary artery disease, myocardial infarction, ischemic stroke, systemic embolism, valvular heart disease, diabetes, hypertension, or dyslipidemia (n = 2,708). The final analysis included 10,003 participants (Figure 1A).

**Figure 1:**
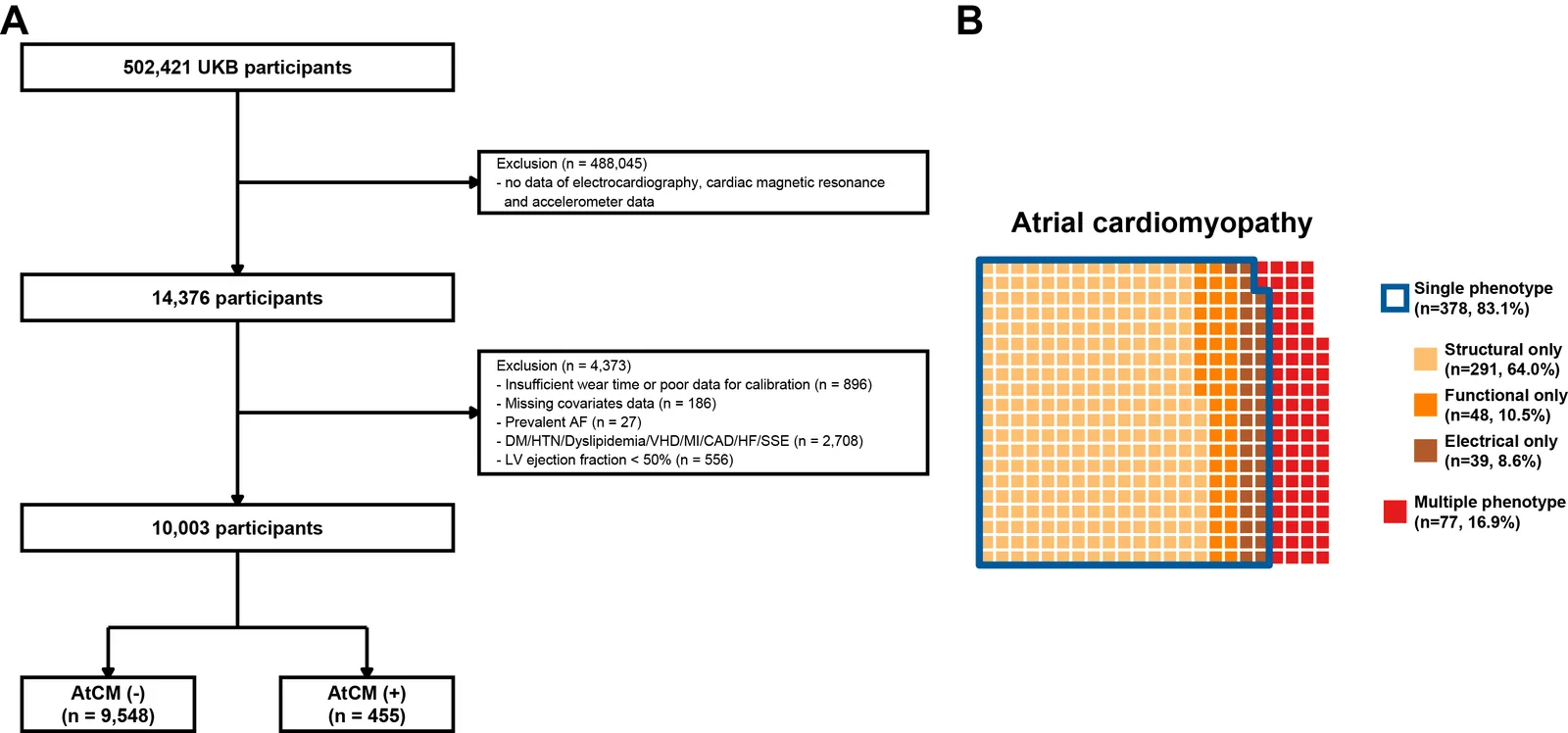
Study Flowchart and Distribution of Atrial Cardiomyopathy Phenotypes (A) Flowchart of the study population selection process and (B) distribution of AtCM phenotypes among the study population. AF = atrial fibrillation; AtCM = atrial cardiomyopathy; CAD = coronary artery disease; DM = diabetes mellitus; HF = heart failure; HTN = hypertension; LV = left ventricular; MI = myocardial infraction; SSE = stroke or systemic embolism; UKB = UK biobank; VHD = valvular heart disease.

This study was conducted in accordance with the Declaration of Helsinki and approved by the Institutional Review Board of the Kangbuk Samsung Hospital (2026-01-022). The current study was conducted using the UK Biobank resource under application No. 77793, and written informed consent was obtained from all participants.

### Definition of atrial cardiomyopathy

Because histological samples were unavailable, AtCM was defined using a multimodal clinical framework integrating structural, functional, and electrical phenotypes derived from CMR and ECG, based on clinical consensus statements and prior literature.^2^^,^^8^^,^^15^ The structural phenotype was defined as left atrial (LA) dilation based on LA volumes indexed to body surface area, with indexed LA maximum volume (LA volume index [LAVI]-max) >60 mL/m^2^ or indexed LA minimum volume (LAVI-min) >30 mL/m^2^. The functional phenotype was defined as an LA emptying fraction <45%. The electrical phenotype was defined by prolonged P-wave duration (>120 ms) and abnormal P-wave axis (<0° or >75°). P-wave indices were derived from automated analyses of resting 12-lead ECGs recorded concurrently with the initial imaging substudy session. Participants meeting any of these structural, functional, or electrical phenotypes were classified into the AtCM (+) group, whereas those without these phenotypes were classified as the AtCM (−) group.

### Device-measured physical activity

Physical activity was objectively assessed using a wrist-worn triaxial accelerometer (Axivity AX3; Axivity Ltd) programmed to record acceleration data at 100 Hz with a dynamic range of ±8 g. Participants were instructed to wear the device on their dominant wrist for 7 consecutive days. Raw accelerometer data were processed using established data-reduction protocols.^13^

Signal processing utilized a validated open-source machine-learning scheme to classify 24-hour movement behaviors into distinct states: sleep, sedentary behavior, light-intensity physical activity, and MVPA.^16^ MVPA was defined as time spent at acceleration magnitudes ≥100 mg, corresponding to an intensity ≥3 metabolic equivalents of task. The primary exposure variable for this analysis was daily MVPA duration (minutes/day).

### Covariates and outcomes ascertainment

Covariate data were obtained from the imaging visit, where available, or from the nearest preceding assessment. Sociodemographic factors collected included age, sex, and the Townsend deprivation index. Anthropometric and clinical measurements included body mass index (BMI), systolic blood pressure (SBP), and diastolic blood pressure, with obesity defined as BMI ≥30 kg/m^2^. Lifestyle factors consisted of smoking status and alcohol consumption, both categorized as never, former, or current. Comorbidities and outcomes were ascertained from linked hospital inpatient records using International Classification of Diseases, 10th edition codes and self-reported medical history (Supplemental Table 1). The primary outcome was incident AF. Participants were followed from the date of imaging substudy assessment until the first AF occurrence, death from any cause, or the end of the administrative follow-up period, whichever occurred first.

### Statistical analysis

Baseline characteristics were compared between the AtCM (−) and (+) groups using the Mann-Whitney U test for continuous variables and the chi-square test for categorical variables. Continuous variables are presented as medians with IQRs, and categorical variables as numbers with percentages. Incidence rates of AF were calculated as the number of events per 1,000 person-years. Cumulative incidence curves for incident AF were constructed using the Kaplan-Meier method, and between-group differences were assessed using the log-rank test.

Multivariable cox proportional hazards regression models were used to estimate HRs and 95% CIs. First, to establish baseline risk, we assessed the prognostic association between AtCM status and incident AF. Subsequently, we investigated the association between MVPA and incident AF. Specifically, to evaluate whether the association between physical activity and AF risk was modified by atrial substrate status, we tested the interaction term between MVPA and AtCM status. The primary models were adjusted for potential confounders, including age, sex, BMI, SBP, and LV mass index. As detailed in the exclusion criteria, participants with missing covariate data (n = 186) were excluded. Therefore, the final models were based on a complete-case analysis approach without imputation. The proportional hazard assumption was assessed using Schoenfeld residuals, and no violation was observed in the main analysis.

Potential nonlinearity in the association was assessed using restricted cubic splines. Because the test for nonlinearity indicated no significant deviation from linearity (*P* for nonlinearity = 0.660), the association was modeled as linear. To quantify clinical relevance, we estimated the absolute risk reduction (ARR) associated with MVPA and evaluated the magnitude of risk reduction stratified by AtCM status. Given the potential influence of demographic and metabolic profiles on atrial substrate and remodeling, we performed subgroup analyses specifically among participants with AtCM. Analyses were stratified by age (<65 vs ≥65 years), sex (female vs male), and BMI (<25 vs ≥25 kg/m^2^) to determine whether the association between MVPA and incident AF was modified by these profiles.

Sensitivity analyses were also performed to assess the robustness of our findings. These included the use of a Fine-Gray model^17^ to account for all-cause death as a competing risk, followed by an analysis adjusting for the Cohorts for Heart and Aging Research in Genomic Epidemiology (CHARGE)-AF score^18^ to control for the comprehensive profile of clinical risk factors. We then performed the analyses after excluding specific subgroups, including participants in the highest decile of MVPA, those with SBP <130 mmHg, and those with relatively preserved LA function, defined as an emptying fraction exceeding the cohort median.

To minimize reverse causality, we subsequently performed a landmark analysis excluding AF events within the first 2 years of follow-up. Finally, the model was additionally adjusted for an AF polygenic risk score to assess independence from genetic susceptibility. All statistical analyses were performed using R software (version 4.1.2; R Foundation for Statistical Computing). A 2-sided *P* value <0.05 was considered statistically significant.

## Results

### Baseline characteristics

Overall, 10,003 participants were included in the final analysis, of whom 455 (4.5%) met the AtCM definition criteria. Most participants with AtCM (83.1%, n = 378) exhibited a single phenotype, suggesting isolated atrial changes, whereas 16.9% (n = 77) had multiple phenotypes, indicative of advanced atrial remodeling. Among these phenotypes, the structural phenotype was the most prevalent component, and the detailed distribution of specific phenotypes is shown in Figure 1B.

Baseline characteristics stratified by AtCM status are summarized in Table 1. Participants with AtCM were older and comprised a higher proportion of females than those without AtCM. They also had higher baseline SBP, whereas BMI and the prevalence of obesity were comparable between the 2 groups.

**Table 1:** Characteristics of Study Participants

	AtCM (−) (n = 9,548)	AtCM (+) (n = 455)	*P* Value
Age, years	63 (56, 68)	66 (59, 71)	<0.001
Sex, n (%)			<0.001
Male	5,615 (58.8)	228 (50.1)	
Female	3,933 (41.2)	227 (49.9)	
White ethnicity, n (%)	9,331 (97.7)	441 (96.9)	0.339
Townsend deprivation index	−2.6 (−3.9, −0.6)	−2.7 (−4.0, −0.6)	0.300
Systolic blood pressure, mmHg	131.0 (120.5, 143.5)	136.0 (124.0, 146.5)	<0.001
Diastolic blood pressure, mmHg	80.0 (73.5, 86.5)	79.0 (72.5, 85.0)	0.084
Body mass index, kg/m^2^	25.3 (23.2, 27.9)	25.2 (22.7, 27.8)	0.050
Obesity, n (%)	1,131 (11.8%)	41 (9.0%)	0.078
Smoking status, n (%)			0.769
Never	6,219 (65.1)	290 (63.7)	
Former	3,017 (31.6)	151 (33.2)	
Current	312 (3.3)	14 (3.1)	
Alcohol consumption, n (%)			0.197
Never	294 (3.1)	14 (3.1)	
Former	286 (3.0)	7 (1.5)	
Current	8,968 (93.9)	434 (95.4)	
24-hr movement behavior, hour			
Sleep	8.7 (8.1, 9.4)	8.5 (7.8, 9.2)	<0.001
Sedentary behavior	9.4 (8.2, 10.5)	9.2 (8.1, 10.4)	0.188
Light intensity physical activity	5.0 (4.0, 6.1)	5.0 (4.0, 6.2)	0.542
Moderate-to-vigorous intensity physical activity	0.6 (0.3, 1.0)	0.8 (0.5, 1.3)	<0.001
Electrocardiography parameter			
P-wave duration, msec	98 (88, 108)	102 (90, 114)	<0.001
P-wave axis, °	54 (38, 66)	58 (42, 72)	<0.001
Cardiac magnetic resonance scan parameter			
LAVI-max, mL/m^2^	37.4 (30.9, 44.2)	61.9 (57.1, 65.9)	<0.001
LAVI-min, mL/m^2^	14.2 (10.7, 17.9)	29.1 (24.5, 33.0)	<0.001
LAEF, %	61.8 (57.4, 66.6)	51.7 (44.6, 58.7)	<0.001
LV mass index, g/m^2^	43.3 (38.7, 49.3)	49.3 (43.0, 57.0)	<0.001
Charlson comorbidity index	0 (0, 0)	0 (0, 0)	0.432
Hospital frailty risk score	0 (0, 0)	0 (0, 0)	0.860
CHARGE-AF score (5-year risk), %	2.1 (1.0, 3.6)	2.9 (1.4, 4.7)	<0.001

Values are presented as medians (IQR) or numbers (%).

AF = atrial fibrillation; AtCM = atrial cardiomyopathy; CHARGE = Cohorts for Heart and Aging Research in Genomic Epidemiology; LAEF = left atrium emptying fraction; LAVI = left atrium volume index; LV = left ventricular; LVMI = left ventricle mass index.

The AtCM (+) group was characterized by larger LAVI (median LAVI-max: 61.9 vs 37.4 mL/m^2^; median LAVI-min: 29.1 vs 14.2 mL/m^2^), impaired LA mechanics (median LA emptying fraction: 51.7% vs 61.8%), prolonged P-wave duration (median 102 vs 98 ms), and abnormal P-wave axis (median 58° vs 54°) at baseline. The detailed distributions of CMR and ECG indices are shown in Supplemental Figures 1 and 2.

Regarding 24-hour movement behavior, the AtCM (+) group engaged in higher daily MVPA (median 0.8 vs 0.6 h) and lower sleep duration (median 8.5 vs 8.7 h). Although both groups had low comorbidity and frailty scores, the predicted 5-year AF risk (CHARGE-AF score) was significantly higher in the AtCM (+) group.

### Prognostic significance of atrial cardiomyopathy for atrial fibrillation risk

During a median follow-up of 3.2 years (IQR: 2.0-4.7), 85 incident AF events occurred (2.51 per 1,000 person-years). Participants with AtCM had a significantly higher risk of new-onset AF than did those without (incidence rate: 11.6 vs 2.10 per 1,000 person-years; HR: 3.01; 95% CI: 1.69-5.37; *P* < 0.001). There was a graded relationship between the number of AtCM phenotypes and AF risk. Although the risk increase was less pronounced and statistical marginal among participants with a single phenotype (HR: 2.03; 95% CI: 0.98-4.22; *P* = 0.057), it was remarkably increased in those with multiple phenotypes (HR: 6.87; 95% CI: 3.13-15.1; *P* < 0.001). The linear trend across the number of AtCM phenotypes (none, single, and multiple) was significant (*P* for trend <0.001) (Supplemental Table 2).

### Association of physical activity according to the presence of atrial cardiomyopathy

In the overall study population, higher MVPA was associated with a significant reduction in incident AF risk (HR per 10-minute MVPA increase: 0.91; 95% CI: 0.85-0.98; *P* = 0.007). We further investigated whether this association was modified by AtCM status. Among participants without AtCM, higher MVPA was marginally associated with AF risk reduction (HR per 10-minute MVPA increase: 0.94; 95% CI: 0.87-1.01; *P* = 0.101). Conversely, in participants with AtCM, higher MVPA was significantly associated with a lower AF risk (HR per 10-minute MVPA increase: 0.76; 95% CI: 0.62-0.93; *P* = 0.007). The magnitude of this inverse association was significantly greater in individuals with AtCM (*P* for interaction = 0.029) (Figure 2).

**Figure 2:**
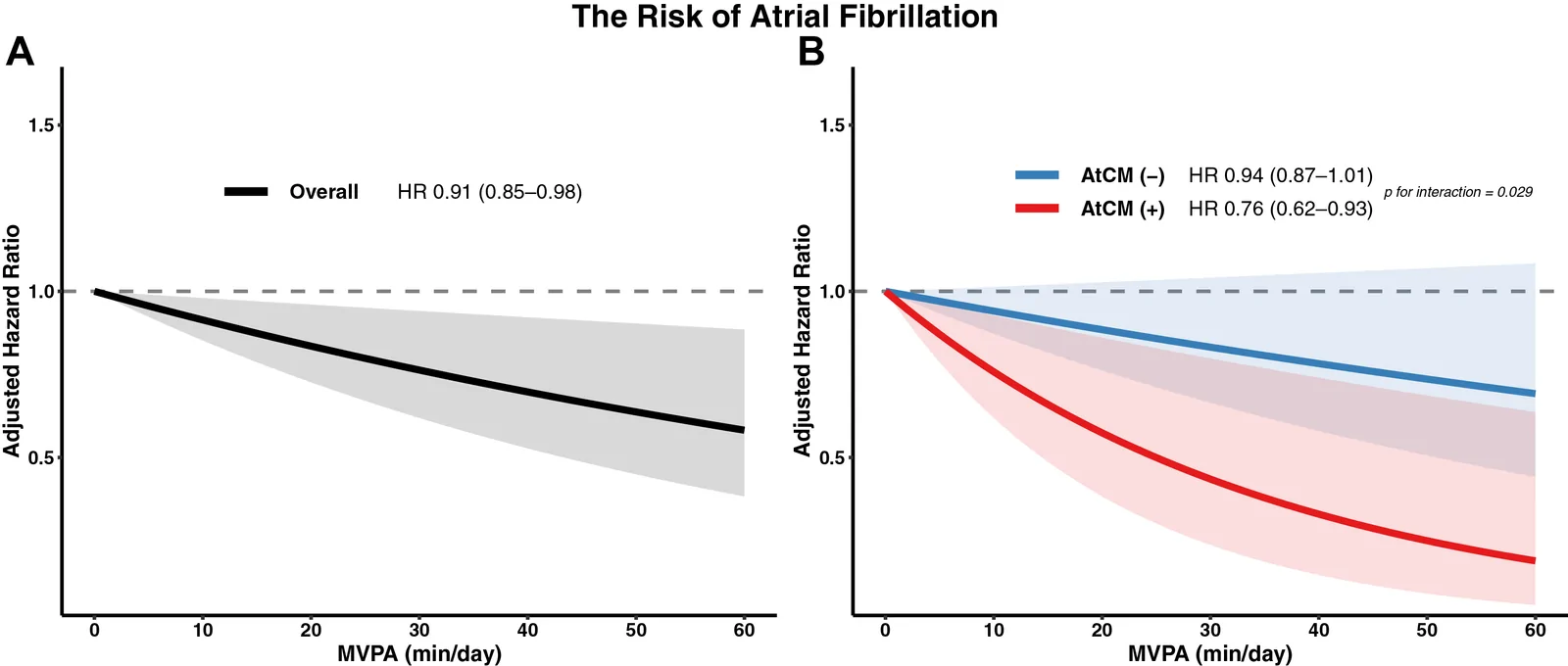
Association Between MVPA and Incident Atrial Fibrillation Risk by AtCM Status (A) Overall population. (B) Stratified by AtCM status. MVPA = moderate-to-vigorous intensity physical activity; other abbreviation as in Figure 1.

Table 2 shows the detailed association between MVPA and incident AF. In the overall population, AF incidence decreased progressively from 2.92 per 1,000 person-years in the lowest MVPA tertile to 2.08 per 1,000 person-years in the highest tertile. Consistently, participants in the highest MVPA tertile had significantly lower AF risk than did those in the lowest tertile (HR: 0.49; 95% CI: 0.28-0.86), reflecting a significant linear trend toward lower risk (*P* for trend = 0.013).

**Table 2:** Association of MVPA With the Risk of Incident AF According to the Presence of AtCM

	MVPA Level	No. of Events/No. at Risk	Incidence Rate (/1,000 PYs)	Unadjusted HR (95% CI)	Model 1^a^ HR (95% CI)	Model 2^b^ HR (95% CI)	*P* for Trend
Overall	Overall (per 10-min increase)	85/10,003	2.51	0.96 (0.90−1.03)	0.94 (0.88−1.01)	0.91 (0.85-0.98)	−
	Tertile 1	34/3,335	2.92	1.00 (Reference)	1.00 (Reference)	1.00 (Reference)	0.013
	Tertile 2	28/3,334	2.52	0.87 (0.53-1.43)	0.84 (0.51-1.40)	0.80 (0.48-1.33)	
	Tertile 3	23/3,334	2.08	0.72 (0.42-1.22)	0.61 (0.36-1.05)	0.49 (0.28-0.86)	
AtCM (−)	Overall (per 10-min increase)	68/9,548	2.10	0.98 (0.92−1.05)	0.96 (0.89−1.03)	0.94 (0.87-1.01)	−
	Tertile 1	26/3,235	2.30	1.00 (Reference)	1.00 (Reference)	1.00 (Reference)	0.112
	Tertile 2	22/3,188	2.06	0.91 (0.51-1.60)	0.86 (0.49-1.52)	0.83 (0.46-1.48)	
	Tertile 3	20/3,125	1.93	0.84 (0.47-1.51)	0.69 (0.38-1.25)	0.61 (0.33-1.13)	
AtCM (+)	Overall (per 10-min increase)	17/455	11.6	0.89 (0.64-0.94)	0.80 (0.66−0.98)	0.76 (0.62-0.93)	−
	Tertile 1	8/100	24.3	1.00 (Reference)	1.00 (Reference)	1.00 (Reference)	0.005
	Tertile 2	6/146	13.3	0.54 (0.19-1.56)	0.65 (0.22-1.87)	0.53 (0.18-1.56)	
	Tertile 3	3/209	4.40	0.20 (0.06-0.75)	0.24 (0.08-0.95)	0.14 (0.04-0.60)	

Tertiles of MVPA were determined based on the overall population (N = 10,003) to ensure a standardized baseline for comparison.

AF = atrial fibrillation; MVPA = moderate-to-vigorous intensity physical activity; PYs = person-years; other abbreviation as in Table 1.

a Adjusted for age and sex.

b Adjusted for age, sex, body mass index, systolic blood pressure, and LV mass index.

When stratified by AtCM status, AF incidence among participants without AtCM decreased numerically across MVPA levels, ranging from 2.30 to 1.93 per 1,000 person-years. However, the risk reduction in the highest tertile did not reach statistical significance compared with that in the lowest tertile (HR: 0.61; 95% CI: 0.33-1.13), showing a marginal trend toward an inverse assocication (*P* for trend = 0.112). Conversely, AF incidence among participants with AtCM declined substantially with increasing MVPA levels, decreasing from 24.3 per 1,000 person-years in the lowest tertile to 4.40 per 1,000 person-years in the highest tertile. This corresponds to an 86% reduction in AF risk in the highest tertile compared with that in the lowest (HR: 0.14; 95% CI: 0.04-0.60), with a significant graded inverse association (*P* for trend = 0.005). The inverse association of MVPA appeared consistent across AtCM phenotypes (Supplemental Table 3).

Cumulative incidence curves were constructed for participants with high vs low MVPA, dichotomized at the median value (0.62 h). Figure 3 illustrates the cumulative AF incidence by MVPA level. When stratified by AtCM status, the cumulative AF incidence among participants without AtCM (Figure 3A) was comparable between the high- and low-activity groups (log-rank *P* = 0.478). Conversely, among those with AtCM (Figure 3B), the cumulative incidence of AF differed significantly, with the high-activity group showing a lower cumulative AF incidence than did the low-activity group (log-rank *P* < 0.001).

**Figure 3:**
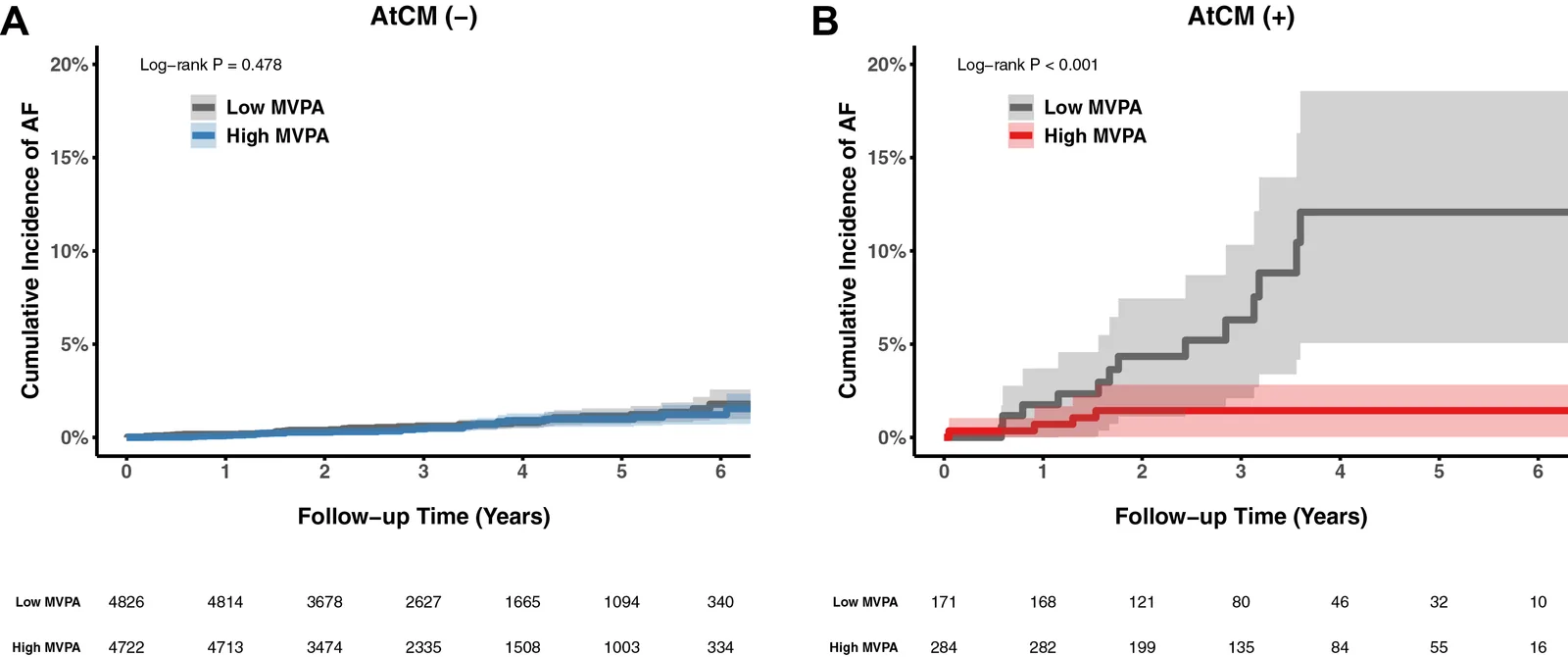
Cumulative Incidence of AF by MVPA Level Participants were stratified into high and low MVPA groups, dichotomized at the cohort median of 0.62 hours. (A) Participants without AtCM; (B) participants with AtCM. Abbreviations as in Figures 1 and 2.

Finally, the clinical relevance of these findings was quantified by estimating the ARR associated with higher MVPA (Figure 4). The magnitude of the inverse association of MVPA was significantly greater among individuals with AtCM. Specifically, for every 10-minute increase in daily MVPA, the estimated ARR was −2.82 (95% CI: −4.44 to −0.84) per 1,000 person-years in the AtCM group, compared with only −0.13 (95% CI: −0.27 to 0.03) in those without AtCM. The difference in ARR between the 2 groups was statistically significant (difference: −2.66; 95% CI: −4.32 to −0.73; *P* = 0.013), highlighting the substantial potential of physical activity as a targeted upstream intervention specifically for this high-risk substrate population.

**Figure 4:**
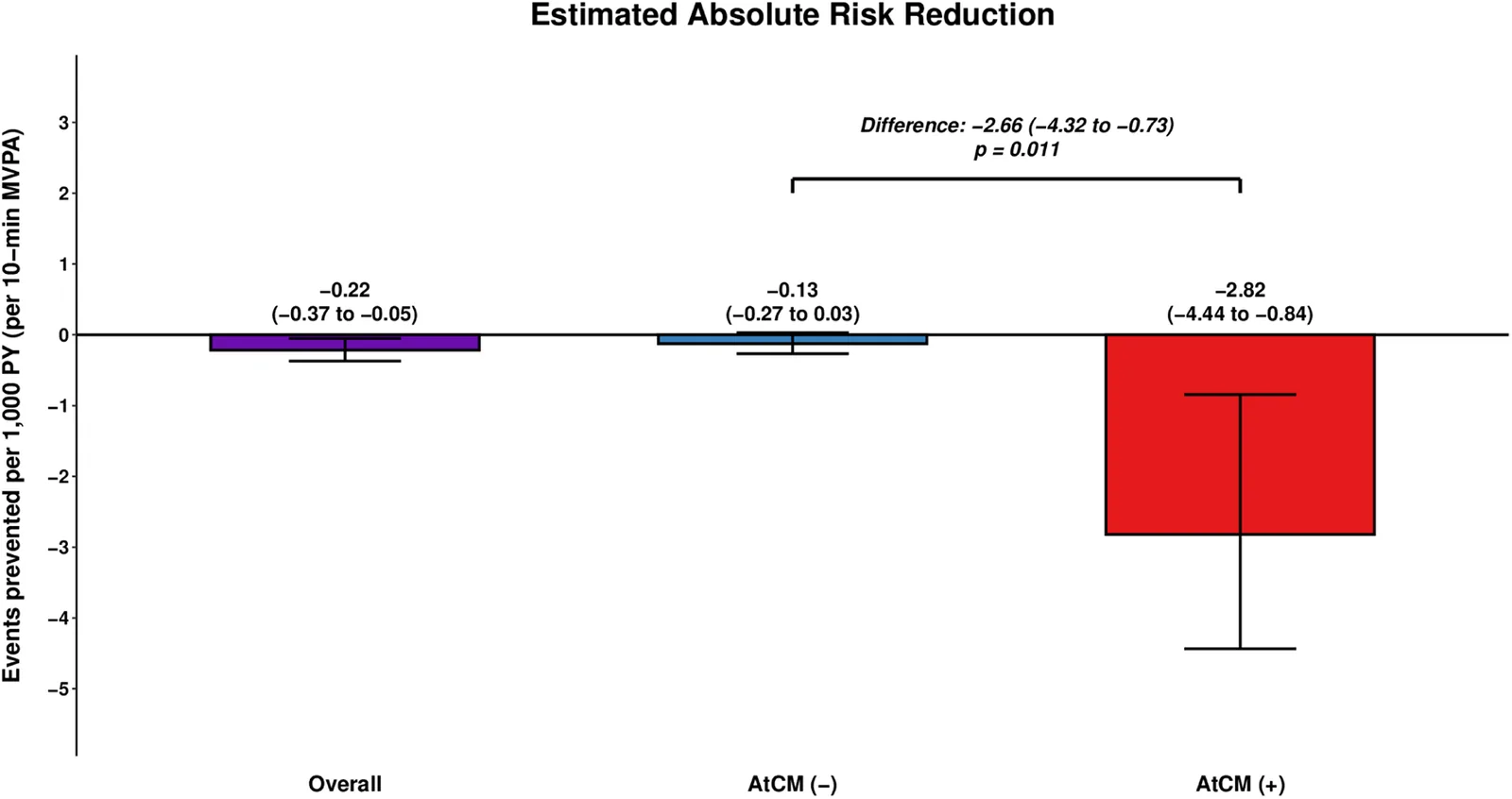
Absolute Atrial Fibrillation Risk Reduction by AtCM Status Estimated absolute risk reduction of AF events per 1,000 person-years associated with a 10-minute increase in MVPA according to the presence of AtCM. PY = person-years; other abbreviation as in Figures 1 and 2.

### Subgroup and sensitivity analyses

In subgroup analyses performed among participants with AtCM, the favorable association of MVPA with incident AF remained consistent across clinical characteristics. No significant interaction was observed by age (*P* for interaction = 0.389), sex (*P* for interaction = 0.192), or BMI (*P* for interaction = 0.094) (Supplemental Figure 3). The association with reduced AF risk in participants with AtCM remained consistent across various sensitivity analyses (Supplemental Table 4). First, in a competing risk analysis accounting for all-cause death, MVPA was significantly associated with a reduced AF risk in the AtCM (+) group (HR: 0.76; 95% CI: 0.63-0.91; *P* = 0.003), with a significant interaction between AtCM status and MVPA (*P* for interaction = 0.016).

Second, even after adjustment for predicted clinical risk using the CHARGE-AF score, the inverse association remained robust in the AtCM (+) group (HR: 0.77; 95% CI: 0.63-0.93; *P* = 0.007), whereas it was marginal in the AtCM (−) group (HR: 0.94; 95% CI: 0.88-1.01; *P* = 0.117; *P* for interaction = 0.013). Third, the results remained robust after excluding participants in the top 10th MVPA percentile (HR: 0.72; 95% CI: 0.56-0.92; *P* = 0.010). Fourth, consistent results were observed when participants with SBP <130 mmHg were excluded (HR: 0.73; 95% CI: 0.56-0.92; *P* = 0.010).

Fifth, the association remained significant in the subgroup with impaired LA function (LA emptying fraction below the cohort median of 61.4%; HR: 0.75; 95% CI: 0.60-0.94; *P* = 0.012). Sixth, when excluding AF events occurring within the first 2 years of follow-up to minimize reverse causality, the association remained directionally consistent (HR: 0.70; 95% CI: 0.45-1.08), although statistical significance was attenuated (*P* = 0.060). Finally, the results remained consistent after additional adjustment for genetic predisposition using the AF polygenic risk score (HR: 0.74; 95% CI: 0.60-0.92; *P* = 0.007).

## Discussion

In this large-scale prospective study of 10,003 participants, we identified several major findings regarding the role of objectively measured MVPA in the transition from AtCM to AF (Central Illustration). First, AtCM, defined through a multimodal integration of CMR imaging and ECG indices, was identified in 4.5% of the community-based population, with most affected individuals exhibiting a single phenotype. Second, our data suggest that AtCM may carry prognostic implications that are not fully captured by traditional clinical risk models. Using the CHARGE-AF score, we estimated a relatively low median 5-year risk of 2.9% for the AtCM (+) group; however, the observed incidence rate translated to an approximate 5-year risk of 5.9% (11.6 per 1,000 person-years), which was more than double the predicted risk. This discrepancy indicates that risk assessment based solely on clinical factors may be limited in predicting atrial remodeling-driven arrhythmogenic risk. Furthermore, consistent with a recent secondary analysis from the EAST-AFNET 4 trial,^19^ which demonstrated that advanced stages of AtCM are associated with worse clinical outcomes, we observed a profound graded relationship where the presence multiple AtCM phenotypes markedly amplified AF risk compared to a single phenotype. This underscores the prognostic importance of quantifying AtCM severity.

**Central Illustration:**
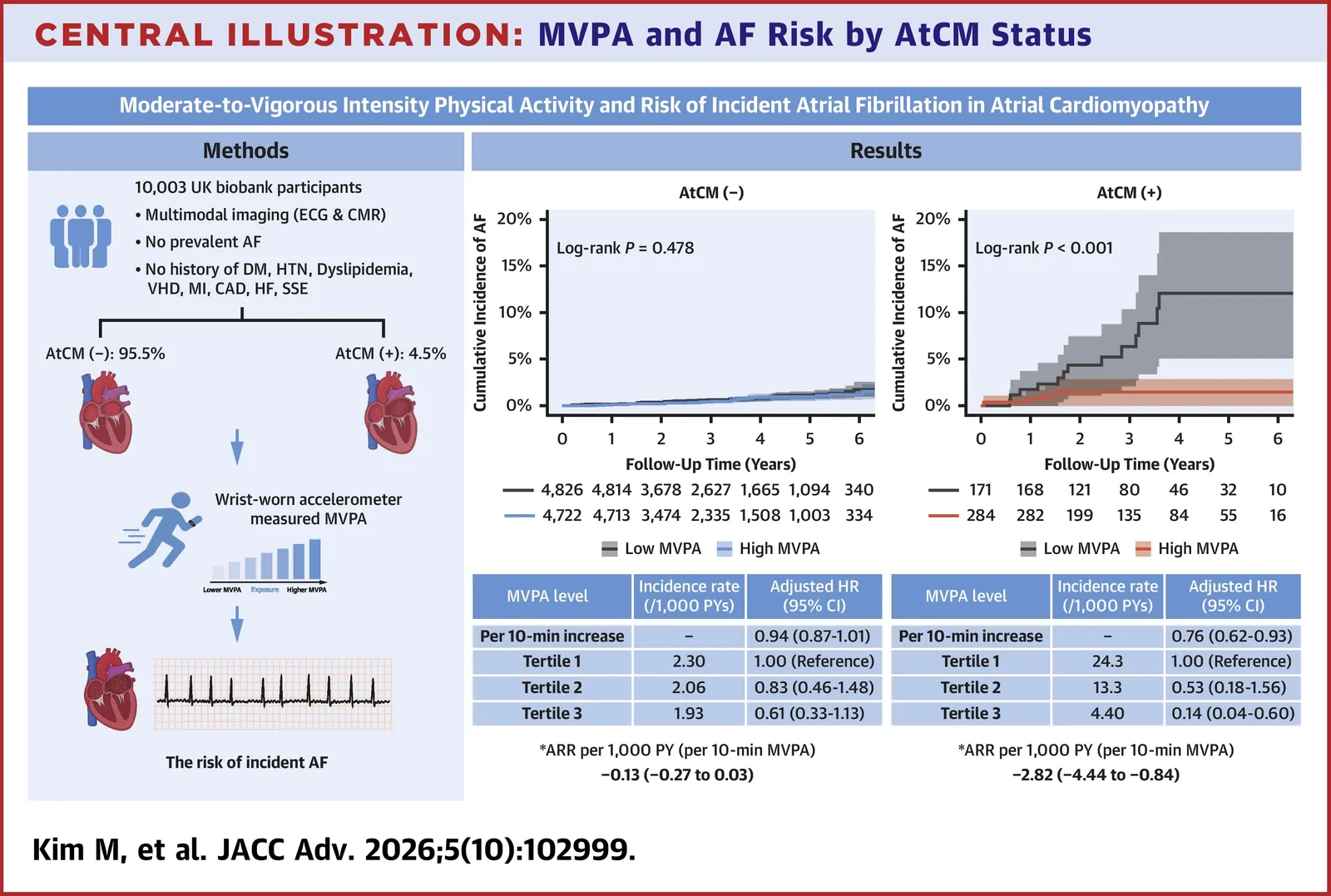
MVPA and AF Risk by AtCM Status In this analysis of 10,003 participants, the inverse association between MVPA and incident atrial fibrillation AF was significantly modified by the underlying atrial substrate. This inverse association was highly pronounced in patients with AtCM. AF = atrial fibrillation; ARR = absolute risk reduction; AtCM = atrial cardiomyopathy; CAD = coronary artery disease; CMR = cardiovascular magnetic resonance; DM, diabetes mellitus; ECG = electrocardiogram; HF = heart failure; HTN = hypertension; MI = myocardial infraction; MVPA = moderate-to-vigorous intensity physical activity; PY = person-year; SSE = stroke or systemic embolism; VHD = valvular heart disease.

Third, we demonstrated that the inverse association between MVPA and AF risk was significantly modified by AtCM status. Although the association with reduced AF risk was marginal in participants without AtCM, it was robust and substantial in those with AtCM, characterized by a steeper linear trend toward lower risk. In terms of absolute clinical relevance, we estimated that a 10-minute increase in daily MVPA is associated with and ARR of approximately 2.82 AF events per 1,000 person-years, specifically in participants with AtCM. Consistent with these findings, the associations remained robust across comprehensive sensitivity analyses, including adjustments for genetic risk and the CHARGE-AF score, suggesting that the favorable association of MVPA extends beyond hereditary and traditional clinical risk factors. Collectively, these findings indicate that MVPA may represent an important lifestyle intervention for attenuating the transition from subclinical atrial substrate to clinical arrhythmia.

Our study enables the refinement of the current understanding of AF prevention by moving beyond broad population-based analyses to substrate-specific risk stratification. Earlier large-scale studies based on self-reported questionnaires provided initial insights into the benefits of physical activity but were limited by recall bias.^9^ More recently, Khurshid et al^10^ addressed these methodological limitations using wrist-worn accelerometers in the UK Biobank, establishing a robust inverse association between MVPA and AF in the general population. Although these landmark studies highlighted the overall safety and favorable outcomes associated with MVPA, such unselected-population analyses inherently average risk across a heterogeneous cohort, potentially masking differential associations of MVPA depending on the underlying atrial substrate. By leveraging CMR imaging and ECG to identify this substrate, we observed that the magnitude of the inverse association between MVPA and AF was relatively modest in individuals with healthy atria, but much more pronounced in those with AtCM. This suggests that the generalized benefits reported in prior studies may have been driven largely by the substantial risk reduction in individuals with subclinical atrial remodeling. Therefore, identifying AtCM defines a high-yield population for whom MVPA may serve as a potent and targeted risk-reducing lifestyle strategy.

The pronounced inverse association of MVPA, specifically observed in the AtCM (+) group, further suggests that physical activity may serve as a physiological stabilizer of the diseased atrial substrate. AtCM is fundamentally characterized by interstitial fibrosis, impaired compliance, and mechanical dysfunction, creating a substrate highly susceptible to arrhythmogenesis.^1^^,^^2^ Regular physical activity is known to enhance LV diastolic function and endothelial integrity, which collectively alleviate LA pressure and wall stress.^20^^,^^21^ Although it is well recognized that extreme endurance training can paradoxically increase AF risk through adverse structural remodeling,^20^ recent imaging data demonstrated that routine MVPA within this UK Biobank cohort induces cardiac adaptations that safely remain within normal physiological limits.^22^ Because these adaptations are bounded within physiological ranges, their incremental protective impact is likely marginal in healthy individuals who already possess preserved atrial compliance and functional reserve. Conversely, in patients with established AtCM, predominantly those with the structural phenotype as identified in the present study, MVPA may effectively reduce hemodynamic overload on the compromised atrium. This reduction in wall stress may attenuate the vicious cycle of stretch-induced fibrosis and electrical instability, thereby slowing progression from subclinical remodeling to overt clinical arrhythmia.

These findings may have important implications for primary preventive strategies before AF development. To date, pharmacological upstream therapies targeting neurohormonal pathways, such as renin–angiotensin–aldosterone system inhibitors or mineralocorticoid receptor antagonists, have not shown consistent, strong benefits in preventing AF.^23^^,^^24^ Furthermore, although sodium-glucose cotransporter-2 inhibitors and glucagon-like peptide-1 receptor agonists are known to suppress atrial fibrosis and inflammation,^25^^,^^26^ there is currently insufficient evidence regarding their role in reducing AF risk specifically among patients with AtCM. In this context, our data suggest that MVPA may serve as an effective, nonpharmacological upstream intervention for this high-risk population. The robust inverse association observed in our study indicates that identifying AtCM should not merely serve as a prognostic marker but as a valuable guide for targeted physical activity recommendations. This paradigm shift toward substrate-directed lifestyle modification offers an actionable and cost-effective pathway to mitigate the increasing global burden of AF.

The principal strength of this study lies in the rigorous, objective assessment of physical activity using wrist-worn accelerometers and the precise characterization of the atrial substrate through a multimodal approach that integrates CMR imaging and 12-lead ECG indices as advocated in previous studies, to reliably identify AtCM.^2^^,^^3^^,^^8^^,^^27^ By combining this approach, we were able to investigate the complex interaction between lifestyle and subclinical atrial remodeling that was previously obscured in broad population analyses.

### Study Limitations

However, some limitations warrant consideration. First, the observational design precludes definitive causal inference. It is possible that higher MVPA serves, in part, as a biomarker for a generally favorable pathophysiology or lower unmeasured comorbidity burden. However, the robust inverse association, extensive sensitivity analyses, and biological plausibility support a favorable, independent association. Second, the definition of AtCM, particularly with respect to atrial volumes, may overlap with physiological remodeling observed in highly active individuals, such as the athlete’s heart. However, several lines of evidence suggest our AtCM (+) group represents a predominantly pathological phenotype. The AF incidence rate in the AtCM (+) group was markedly higher than that in the AtCM (−) group, contrary to the benign nature of physiological remodeling. Furthermore, we observed a linear, inverse association between MVPA and AF risk within the AtCM (+) group. Importantly, our AtCM definition incorporates functional parameters, specifically impaired LA emptying fraction.

Third, our phenotypic characterization has inherent limitations. Regarding ECG markers, our definition relied on P-wave duration and axis, potentially overlooking other prognostically relevant indices. Similarly, although our CMR analysis provided robust volumetric and functional data, it did not include tissue characterization sequences, such as late gadolinium enhancement, to directly visualize atrial fibrosis. In addition, comprehensive blood biomarker data reflecting atrial stress or inflammation were not uniformly available for the entire cohort, limiting our ability to explore biochemical correlations. Fourth, from a clinical implementation perspective, the widespread utility of CMR is currently restricted by cost, availability, and logistical constraints. Consequently, applying a CMR-based AtCM definition to routine general-population screening remains challenging, although our findings underscore the importance of identifying this high-risk substrate. Fifth, our reliance on administrative 10th edition codes and self-reported medical history for outcome ascertainment precluded the classification of incident AF patterns. Given that recent evidence highlights the substantial impact of specific AF patterns on cardiovascular outcomes,^28^ the inability to assess whether the inverse association of MVPA varies by AF pattern remains a limitation that warrants future investigation.

Sixth, the median interval between accelerometer assessment and imaging acquisition was 2.97 years (IQR: 1.30-4.02 years). This precludes cross-sectional analysis; however, this temporal separation allows assessment of habitual physical activity levels before the characterization of the atrial substrate. Furthermore, we rigorously excluded participants who developed heart failure, myocardial infarction, valvular heart disease, or stroke/systemic embolism between the 2 assessment time points, minimizing the potential confounding influence of declining health or major intercurrent cardiovascular events on physical activity levels. Seventh, physical activity was assessed using wrist-worn accelerometers, which primarily capture ambulatory movements and inherently underestimate stationary, anaerobic, or resistance-based physical activities. Consequently, the specific cardiovascular effects of these alternative modalities could not be evaluated in this study. Finally, the UK Biobank cohort is prone to healthy volunteer bias and is predominantly of White European ancestry, which may limit generalizability to other populations.

In conclusion, this large-scale prospective study indicates that the inverse association of MVPA with AF is significantly modified by the underlying atrial substrate. These findings support a refined approach to AF prevention that moves beyond generalized lifestyle recommendations toward a substrate-specific strategy. Identifying AtCM may serve not only for risk stratification but also as a valuable guide for targeted physical activity prescription, highlighting MVPA as an effective, nonpharmacological upstream intervention to attenuate the transition from subclinical remodeling to clinical arrhythmia.

## Funding support and author disclosures

This research was supported by grants from the Patient-Centered Clinical Research Coordinating Center (PACEN) funded by the 10.13039/100003625Ministry of Health and Welfare, Republic of Korea (grant no. RS-2026-25520638). Dr Joung has served as a speaker for 10.13039/100004326Bayer, 10.13039/100002491BMS/10.13039/100004319Pfizer, 10.13039/100004319Medtronic, and 10.13039/501100002336Daiichi-Sankyo and has received research funds from 10.13039/100004374Medtronic and 10.13039/100000046Abbott. No fees were received, either directly or personally. All other authors have reported that they have no relationships relevant to the contents of this paper to disclose.
