# Supplementary material for: Moderate-to-Vigorous Intensity Physical Activity and Risk of Incident Atrial Fibrillation in Atrial Cardiomyopathy
Source: JACC Adv. 2026 Aug 28;5(10):102999. doi: 10.1016/j.jacadv.2026.102999 (PMC13699701; doi:10.1016/j.jacadv.2026.102999)

SUPPLEMENT MATERIALS

Moderate-to-Vigorous Physical Activity and Risk of Incident Atrial Fibrillation in Atrial Cardiomyopathy

Min Kim, MD; Pil-Sung Yang, MD; Ho-Gi Chung, MD; Juntae Kim, MD; Daehoon Kim, MD; Han-Joon Bae, MD; Moon-Hyun Kim, MD; Boyoung Joung, MD

Contents

Supplemental Table 1. Definitions used for defining the comorbidities and outcome.

Supplemental Table 2. Association between AtCM and the risk of incident AF, overall and stratified by the number of phenotypes.

Supplemental Table 3. Association of a 10-minute increase in MVPA with the risk of AF according to the presence of AtCM and phenotypes.

Supplemental Table 4. Sensitivity analyses for the association between a 10-minute increase in MVPA and the risk of incident AF according to the presence of AtCM.

Supplemental Figure 1. Distribution of P-wave indices between AtCM (-) and AtCM (+). P-wave duration (A), P-wave axis (B)

Supplemental Figure 2. Distribution of LAVI-max, LAVI-min, and LA emptying fraction between AtCM (-) and AtCM (+). LAVI-max (A), LAVI-min (B) LA emptying fraction (C)

Supplemental Figure 3. Association of a 10-minute increase in MVPA with the risk of incident AF in participants with atrial cardiomyopathy stratified by subgroups.

Supplemental Table 1. Definitions used for defining the comorbidities and outcome.

Comorbidities	Definitions	Used codes or conditions
Hypertension	Defined from UK Biobank self-report or diagnosis* plus treatment	Self-reported non-cancer illness code: 1065, 1072<br>ICD-10: I10, I11, I12, I13, I15<br>Treatment: all kinds of blood pressure-lowering medications
Diabetes mellitus	Defined from UK Biobank self-report or diagnosis*	Self-reported non-cancer illness code: 1220, 1222, 1223, 1521<br>ICD-10: E10, E11, E12, E13, E14
Dyslipidemia	Defined from UK Biobank self-report or diagnosis*	Self-reported non-cancer illness code: 1473<br>ICD-10: E78
Prior myocardial infraction	Defined from UK Biobank self-report or diagnosis*	Self-reported non-cancer illness code: 1075<br>ICD-10: I21, I22, I25.2
Heart failure	Defined from UK Biobank self-report or diagnosis*	Self-reported non-cancer illness code: 1076<br>ICD-10: I11.0, I50, I97.1
Valvular heart disease	Defined from a history of valve surgery or diagnosis* of rheumatic/non-rheumatic mitral stenosis	Procedure codes: K25, K26, K27, K28, K29, K30, K31, K32, K33, K34, K35<br>ICD-10: I05.0, I50.2, I34.2, Z95.2, Z95.3, Z95.4
Ischemic stroke or TIA	Defined from UK Biobank self-report or diagnosis*	Self-reported non-cancer illness code: 1583, I082<br>ICD-10: I63, I64, G45
**Outcome**	**Definitions**	**Used codes or conditions**
Atrial fibrillation	Defined from UK Biobank self-report or diagnosis*	Self-reported non-cancer illness code: 1471, 1483<br>ICD-10: I48

*To ensure accuracy, comorbidities were established based on more than one hospital-inpatient or two outpatient (= primary care in United Kingdom) records of ICD-10 codes in the database.

Supplemental Table 2. Association between AtCM and the risk of incident AF, overall and stratified by the number of phenotypes.

	**No. of events/ No. at risk**	**Incidence rate (/1,000 PYRs)**	**Adjusted[a] HR (95% CI)**	**p value**
AtCM (-)	68/9,548	2.10	Reference	−
AtCM (+)	17/455	11.6	3.01 (1.69–5.37)	<0.001
Single phenotype	9/378	7.30	2.03 (0.98–4.22)	0.057
Multiple phenotype	8/77	35.3	6.87 (3.13–15.1)	<0.001

P for linear trend across the number of AtCM phenotypes (none, single, multiple) was <0.001.
a Adjusted for age, sex, body mass index, systolic blood pressure, LVMI.
Abbreviations: AF, atrial fibrillation; AtCM, atrial cardiomyopathy; CI, confidence interval; HR, hazard ratio; PYR, person-years

Supplemental Table 3. Association of a 10-minute increase in MVPA with the risk of AF according to the presence of AtCM and phenotypes.

	Unadjusted<br>HR (95% CI)	Model 1[a]<br>HR (95% CI)	Model 2[b]<br>HR (95% CI)	p value
AtCM (-)	0.98 (0.92−1.05)	0.96 (0.89−1.03)	0.94 (0.87–1.01)	0.108
AtCM (+)	0.80 (0.65–0.95)	0.82 (0.68−0.98)	0.76 (0.62–0.93)	0.007
Single phenotype	0.79 (0.61−1.01)	0.79 (0.61−1.02)	0.77 (0.59–1.00)	0.049
Multiple phenotype	0.78 (0.57−1.07)	0.81 (0.58−1.13)	0.76 (0.52–1.10)	0.144

a Adjusted for age and sex.
b Adjusted for age, sex, body mass index, systolic blood pressure and LV mass index.
Abbreviations: AF, atrial fibrillation; AtCM, atrial cardiomyopathy; CI, confidence interval; HR, hazard ratio; MVPA, moderate-to-vigorous intensity physical activity

Supplemental Table 4. Sensitivity analyses for the association between a 10-minute increase in MVPA and the risk of incident AF according to the presence of AtCM.

	No. of events/ No. at risk	Incidence rate (/1,000 PYRs)	Adjusted[a] HR (95% CI)	P value	P for interaction
**Accounting for all-cause death as a competing risk (n=10,003)**					
AtCM (-)	68/9,548	2.10	0.94 (0.87–1.02)	0.120	0.016
AtCM (+)	17/445	11.6	0.76 (0.63–0.91)	0.003	
**Adjusting[b] for the CHARGE-AF predicted risk score (n=10,003)**					
AtCM (-)	68/9,548	2.22	0.94 (0.88–1.01)	0.117	0.013
AtCM (+)	17/445	12.3	0.77 (0.63–0.93)	0.007	
**Excludes participants with MVPA levels in the top 10th percentile (n=9,001)**					
AtCM (-)	62/8,628	2.12	0.92 (0.82–1.03)	0.143	0.071
AtCM (+)	16/373	13.5	0.72 (0.56–0.92)	0.010	
**Excludes participants with SBP less than 130 mmHg (n=5,346)**					
AtCM (-)	53/5,052	3.11	0.96 (0.88–1.04)	0.286	0.038
AtCM (+)	12/294	12.7	0.73 (0.56–0.94)	0.016	
**Excludes participants with LA emptying fraction above the median 61.4% (n=5,001)**					
AtCM (-)	35/4,612	2.26	0.89 (0.79–1.01)	0.063	0.129
AtCM (+)	15/389	12.2	0.75 (0.60–0.94)	0.012	
**Excludes participants with newly developed AF occurring within the first 2 years of follow-up (n=7,472)**					
AtCM (-)	37/7,152	1.30	0.91 (0.82–1.01)	0.083	0.060
AtCM (+)	6/320	4.83	0.70 (0.45–1.08)	0.103	
**Additional adjustment for AF polygenic risk score (n=9,766)**					

AtCM (-)	68/9,321	2.15	0.94 (0.87–1.02)	0.074	0.052
AtCM (+)	16/445	11.2	0.74 (0.60–0.92)	0.007	

a Adjusted for age, sex, body mass index, systolic blood pressure and LV mass index.
b Adjusted for CHARGE-AF score, sex, and LV mass index.
Abbreviations: AF, atrial fibrillation; AtCM, atrial cardiomyopathy; CI, confidence interval; HR, hazard ratio; LA, left atrium; MVPA, moderate-to-vigorous intensity physical activity; PYR, person-years; SBP, systolic blood pressure

Supplemental Figure 1. Distribution of P-wave indices between AtCM (-) and AtCM (+). P-wave duration (A), P-wave axis (B)

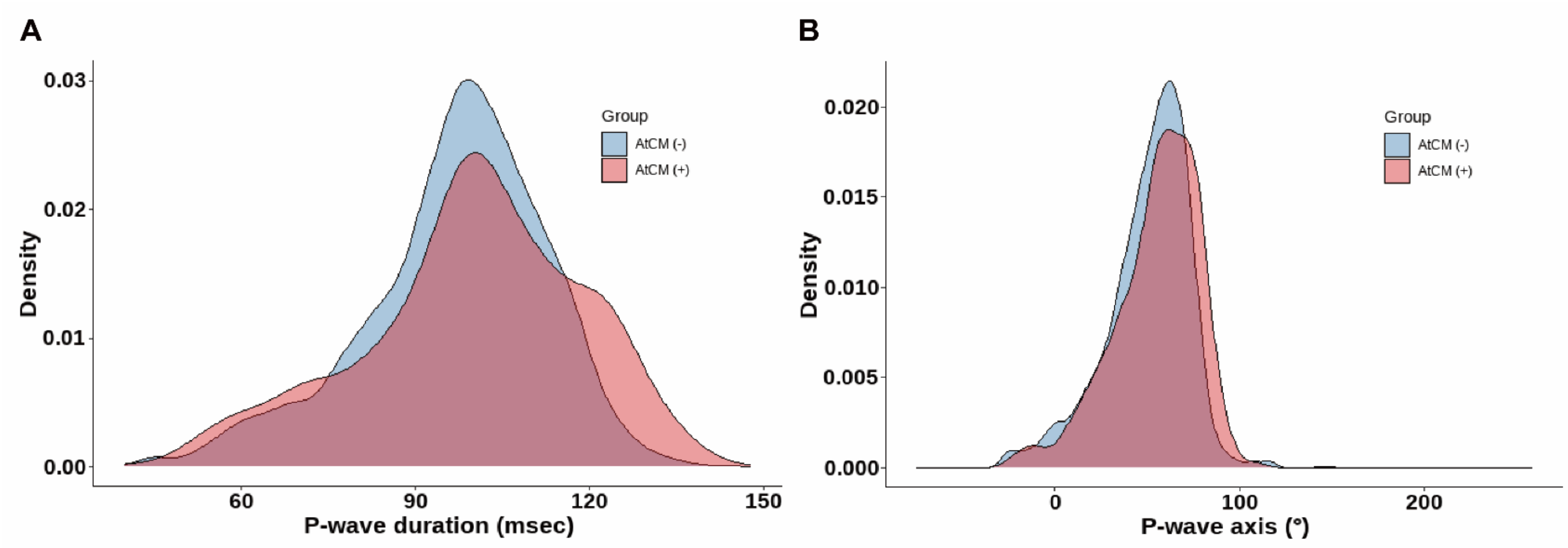

Supplemental Figure 2. Distribution of LAVI-max, LAVI-min, and LA emptying fraction between AtCM (-) and AtCM (+). LAVI-max (A), LAVI-min (B) LA emptying fraction (C)

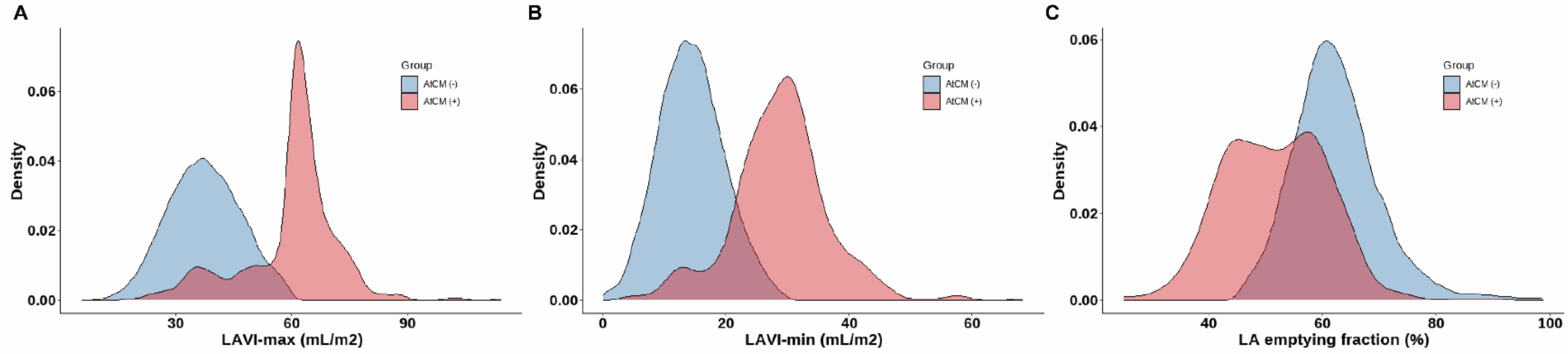

Supplemental Figure 3. Association of a 10-minute increase in MVPA with the risk of incident AF in participants with atrial cardiomyopathy stratified by subgroups.

Subgroup	No. at risk	No. of events	IR (/1,000 PYRs)	HR (95% CI)	
**Age**					*P for interaction = 0.389*
< 65 y	205	3	4.2	0.74 (0.39-1.38)	
≥ 65 y	250	14	19.0	0.80 (0.64-0.99)	
**Sex**					*P for interaction = 0.192*
Female	228	10	13.4	0.66 (0.46-0.96)	
Male	227	7	9.8	0.85 (0.67-1.07)	
**BMI**					*P for interaction = 0.094*
< 25	217	7	10.3	0.64 (0.41-1.02)	
≥ 25	238	10	12.8	0.87 (0.72-1.06)	

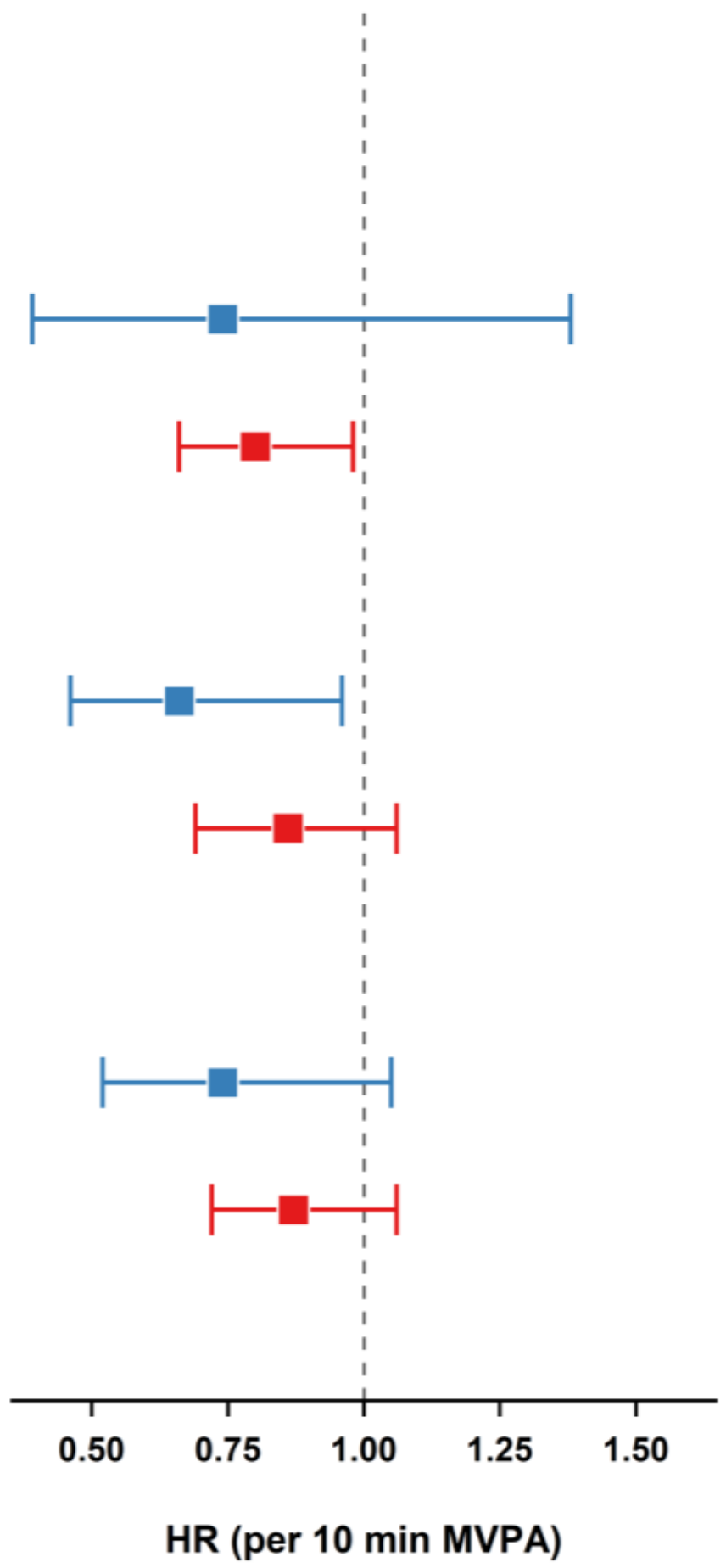

Supplement: Supplementary file 1 [file mmc1.pdf]
